# Integrated genomic analysis of clear cell ovarian cancers identified PRKCI as a potential therapeutic target

**DOI:** 10.18632/oncotarget.19946

**Published:** 2017-08-04

**Authors:** Tsun Yee Tsang, Wei Wei, Hiroaki Itamochi, Rosemary Tambouret, Michael J. Birrer

**Affiliations:** ^1^ Department of Medicine, Massachusetts General Hospital, Boston, Massachusetts, USA; ^2^ Harvard Medical School, Boston, Massachusetts, USA; ^3^ Department of Obstetrics and Gynecology, Tottori University School of Medicine, Yonago, Tottori, Japan

**Keywords:** clear cell ovarian cancer, PRKCI, genomics, microarray, therapeutic target

## Abstract

Clear cell ovarian cancer (CCOC) is an epithelial ovarian cancer histotype with unique pathologic, biologic and clinical features. Despite its worse prognosis than serous ovarian cancer (SOC), the genomic landscape of CCOC is less well defined. Integrated genomic analysis of CCOC allows the identification of potential therapeutic targets to improve the treatment of this tumor. Using comparative genomic hybridization and gene expression profiling, we have screened 12 CCOC cell lines and 40 tumors to identify 45 amplified and overexpressed genes. Pathways analysis of these genes identified 19 genes with cancer-related functions. Of these, PRKCI is one of the most frequently amplified and overexpressed genes and its expression induced cancer cell proliferation and migration/invasion *in vitro* as well as tumor growth *in vivo*. Targeting PRKCI by small molecule inhibitor, sodium aurothiomalate (ATM), significantly reduced the *in vivo* tumor growth and may be a new therapeutic strategy to improve the treatment of CCOC.

## INTRODUCTION

Ovarian cancer is the most lethal of gynecologic cancers in the United States resulting in 14,000 deaths each year [1, 2]. The majority of ovarian cancer arises from fallopian tube or ovarian surface epithelial cells and despite major differences in pathology are all treated with surgery followed by chemotherapy. There are different histological subtypes, including serous, clear cell, endometrioid, and mucinous, and recent efforts have begun to appreciate that each subtype has distinct genetic features, biologic properties and clinical outcome [3].

Clear cell ovarian cancer (CCOC) is the third most common subtype of ovarian cancer, with an estimated incidence of 5 % of all epithelial ovarian malignancies [4, 5]. CCOC has a poorer prognosis than other histotypes in that patients with CCOC have higher risk of recurrence and lower survival rate when compared with patients with serous ovarian cancer. This may be partially due to the fact that these tumors are less responsive to platinum/taxane-based chemotherapy [6, 7]. Therefore, the development of a more effective therapeutic approach specific for CCOC is an unmet clinical need and this will likely require the identification of new therapeutic targets

Recent studies have revealed distinct genomic landscape in CCOC. Unlike serous tumors which has relatively low mutation load (except for *TP53*), CCOC is characterized with various key functional mutations [3]. This includes the most prominent loss-of-function in tumor suppressor *ARID1A* in approximately 50% of cases [8], followed by the gain-of-function in *PI3KCA* and *CTNNB1* conferring hyperactivated PI3K/Akt and Wnt/β-catenin pathway respectively [9]. On the other hand, various cancers are hallmarked by chromosomal structural aberrances such as DNA copy number variation (CNV) which may act as definitive drivers of tumorigenesis. However, due to the rarity of clear cell ovarian cancers, very little is known about the CNV of these tumors. Moreover, previous efforts in the study of CCOC genomics that focused on CNV [10-12] did not explore its association with differential expression or potential biological consequences in CCOC. Therefore, driver genes for this tumor have not been well established and candidate therapeutic targets remain to be identified.

The aims of this study were two-fold: (1) the identification of potential therapeutic target genes through an integrated genomics approach; and (2) a “proof-of-principle” demonstration that genes on the list impact ovarian clear cell cancer biology and can be potential therapeutic targets. Through integrated analyses of high-resolution array comparative genomic hybridization (aCGH) and microarray-based gene expression profiling data generated from CCOC cell lines and patient tumor specimens, we have generated a list of candidate genes with DNA copy number amplification associated with mRNA overexpression. The candidate genes were further screened for important cancer-related functions through bioinformatic annotation. This approach led to the identification of genes that were potential “drivers” for tumorigenesis of clear cell cancers of the ovary, a disease distinctive in clinicopathology and molecular biology to the high-grade serous carcinoma as the most common ovarian cancer subtype [3].

## RESULTS

### Global DNA copy number alterations in CCOC

**Figure 1:**
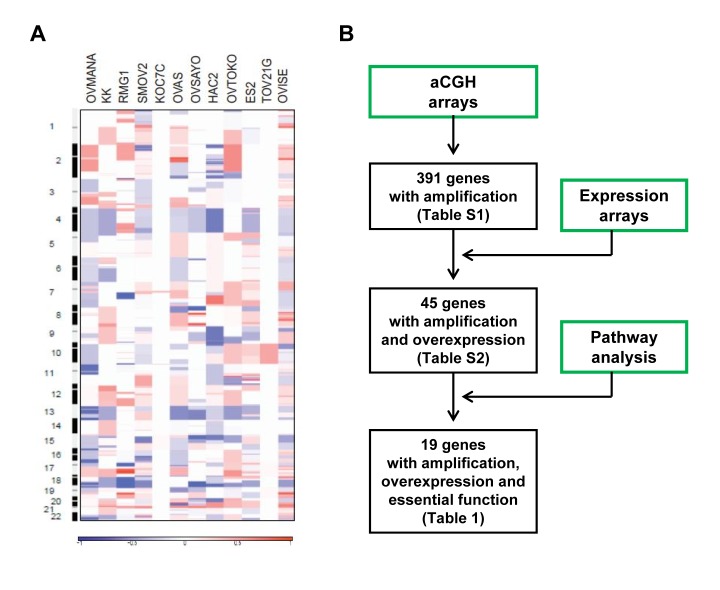
Global genomics analysis of clear cell ovarian cancer **A.** Genome-wide copy number alterations in clear cell ovarian cancer cell lines detected by aCGH. Pseudocolor gradients corresponding to the copy number amplification (red boxes) and deletion (blue boxes) compared with pooled normal samples. **B.** Workflow diagram of integrated analysis on aCGH, expression profiling data and pathway analysis by PathwayStudio 6.0 software.

The genomic DNA copy status of CCOC was investigated by high-resolution aCGH using Agilent human 105K oligonucleotide microarrays on 12 CCOC cell lines. Genomic copy number for each probe was determined by calculating the log2 ratio of median signal intensities of the cell lines and normal reference DNA. A genome-wide view of the copy number variation in the cell lines is shown in Figure 1A. Frequent regions of copy-number alterations were identified using the statistical method Genomic Identification of Significant Targets In Cancer (GISTIC). GISTIC identified 16 amplified regions, which contain 391 genes. Chromosomal locations, frequencies, genomic intervals and number of gene contents are shown in Supplementary Table S1. Minimal common regions for the most frequent copy number gains were at 20q13.2 (10 of 12, 83%), 17q22 (7 of 12, 58%), and 3q26.31 (6 of 12, 50%).

### Integrated genomic analysis identifies amplified and overexpressed genes in COCC

To identify “driver” genes in the 16 amplified regions, we conducted an integrated genomic analysis utilizing gene expression profiling and pathways analysis. The workflow diagram of the integrated analysis is shown in Figure 1B. Gene expression profiles of CCOC were employed to determine overexpressed genes among the list of 391 amplified gene. The gene expression pattern of 10 laser capture micro-dissected CCOC tumor specimens were compared to 10 normal ovarian surface epithelium specimens using Affymetrix U133 plus 2 arrays as reported previously [13]. 2559 genes were found to be differentially regulated as defined by a 1.5-fold or greater difference in expression with a statistical significance of *p* < 0.001. Among the 391 amplified genes, 45 of them were associated with mRNA overexpression (Supplementary Table S2). Secondly, the 45 amplified and overexpressed genes were imported into PathwayStudio 6.0 software to determine genes involved in important biological pathways. Genes involved in cell proliferation, cell growth, apoptosis, cell migration, cell invasion, angiogenesis, DNA replication and DNA repair were identified. Among the 45 amplified and mRNA overexpressed genes, 19 of them were involved in the regulation of cancer-related biological function and are listed in Table 1. The 19 genes identified are not found in the known amplicons in serous ovarian cancer (SOC) [14, 15].

**Table 1:** Potential target genes with gene amplification, overexpression and oncogenic function.

DNA amplification^#^		mRNA overexpression^##^ Fold change (T/N)	Function
Amplification peak	Potential Target Genes		
**20q13.2**	**ZNF217**	**6.28**	Cell proliferation, transcription regulation, apoptosis inhibition
**17q22**	**APPBP2**	**3.92**	Meiosis regulation
	**TMEM49**	**2.47**	Cell motility, cell invasion, apoptosis inhibition
**3q26.31**	**PRKCI**	**6.70**	Cell growth, cell migration and invasion, apoptosis inhibition
	**ECT2**	**4.72**	Cell growth and proliferation
**19q12**	**GPI**	**2.72**	Cell proliferation, glucose metabolism, DNA replication
	**PSENEN**	**4.50**	Cell proliferation
	**CEBPG**	**3.03**	Transcription regulation
**8q11.23**	**MCM4**	**2.15**	Cell growth , cell cycle, DNA replication
	**PRKDC**	**3.73**	DNA repair, cell proliferation
	**UBE2V2**	**4.14**	DNA repair and replication
**2q13**	**PAX8**	**7.21**	Cell division, cell proliferation, apoptosis inhibition
**8q24.3**	**PTK2**	**2.87**	Cell invasion, cell growth, angiogenesis, DNA repair and replication
**1q44**	**ADSS**	**2.63**	Purine metabolism
	**ARID4B**	**3.52**	Cell growth, translation regulation
	**FH**	**3.13**	Cell growth, purine metabolism
	**HNRNPU**	**3.07**	Chromatin remodeling, cell proliferation
	**MTR**	**3.08**	DNA replication
**4p16.3**	**FGFR3**	**12.9**	Cell growth, apoptosis inhibition, angiogenesis

# Copy number variation determined by aCGH on 12 CCOC cell lines.

## mRNA differentially expression between 10 CCOC tumor specimens (T) and 10 normal ovarian surface epithelium specimens (N) by expression microarray analysis.

### Identification of PRKCI as a potential driver and therapeutic target for CCOC

**Figure 2:**
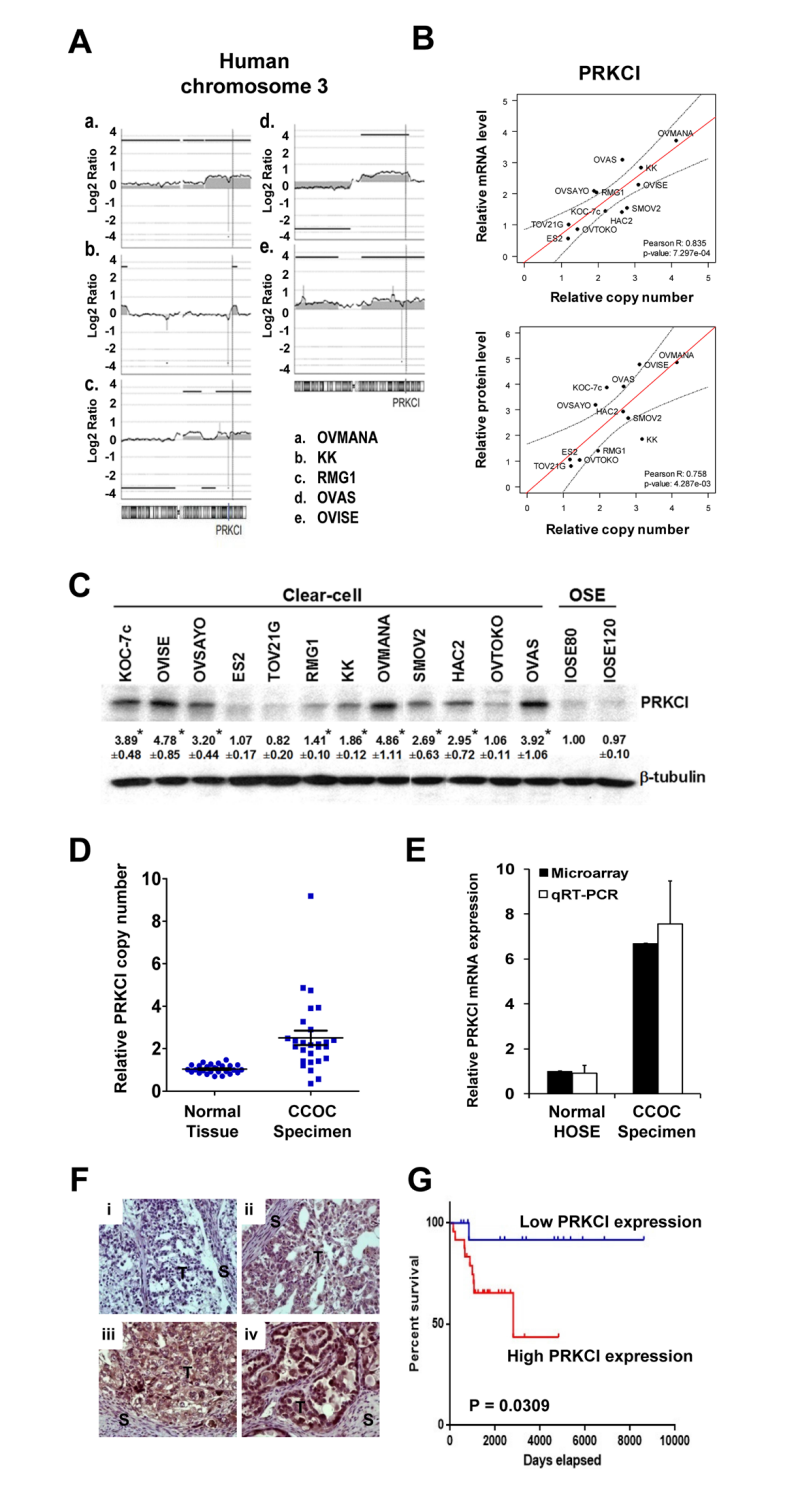
PRKCI amplification and overexpression in CCOC cells and patient specimens **A.** Copy number alterations in human chromosome 3 of five CCOC cell lines indicate amplification at 3q26 region, containing PRKCI gene. **B.** Correlation between PRKCI DNA copy number, mRNA expression and protein expression in 12 clear cell ovarian cancer cell lines. Relative PRKCI copy number, mRNA expression level and protein expression level of 12 clear cell ovarian cancer cell lines were determined by qPCR, qRT-PCR and Western blot respectively. The correlation was determined by Pearson correlation coefficient. **C.** PRKCI protein expression level of 12 CCOC cell lines and 2 normal ovarian surface epithelium cell lines (IOSE-80 and IOSE-120) as determined by Western blot analysis. **D.** Copy number variation qPCR on 27 CCOC tumor specimen. Relative PRKCI copy number was calculated by comparing normal and clear cell cancer specimen with pooled normal reference genomic DNA. **E.** Microarray and qRT-PCR data of PRKCI mRNA expression level by comparing 10 CCOC tumor specimens with 10 normal ovarian surface epithelium samples. **F.** Immunohistochemical staining of PRKCI protein expression in an additional 40 CCOC tumor tissues. Representative pictures are shown to demonstrate PRKCI intensity of (i) 0, (ii), 1+, (iii) 2+, (iv) 3+. Original magnification x200. **G.** Kaplan-Meier estimates of survival of the 40 CCOC patients according to the PRKCI expression level in cancer tissue with immunohistochemical staining. Analysis was done by median cut with the p-value of log-rank test. Blue line, samples with low PRKCI ( < median); red line, samples with high PRKCI (≥median). Patients with high expression of PRKCI showed lower percentage survival compared with patients with low expression of PRKCI.

To validate our integrated analysis, we selected one gene for “proof of principle” evaluation and characterized its biologic and clinical impact. Among the list of 19 genes, PRKCI is the most highly overexpressed gene within the three most frequently amplified chromosomal regions. Amplification of PRKCI, which is located at 3q26, was shown in the aCGH profiles of 5 CCOC cell lines (Figure 2A). Amplification of PRKCI was not found in serous ovarian cancer according to TCGA data [15]. PRKCI amplification was validated by qPCR on the same 12 CCOC cell lines. Elevated PRKCI copy number was found in 9 CCOC cell lines when compared with normal reference DNA (defined as 1 in Figure 2B). Overexpression of PRKCI was found in 9 CCOC cell lines when compared with normal ovarian surface epithelium cell lines (IOSE80 and IOSE120, the average level defined as 1 in Figure 2B). A strong correlation was found between the relative PRKCI copy number and PRKCI mRNA expression (Pearson’s *r* = 0.8348, *p* = 0.0007), suggesting mRNA overexpression of PRKCI is due to amplification (Figure 2B). PRKCI protein overexpression was also found in the nine PRKCI amplified and overexpressed CCOC cell lines when compared with the two normal OSE cell lines by Western blot analysis (Figure 2C). Furthermore, the protein levels of PRKCI in CCOC cell lines also significantly correlate with the copy number of PRKCI gene (Pearson’s *r* = 0.7579, *p* = 0.0043). Notably, the amplification and overexpression of PRKCI were found specifically in CCOC cell lines but not in SOC cell lines (Supplementary Figure 1).

### PRKCI is amplified and overexpression in human CCOC and predicts patient survival

To further suggest the clinical relevance of PRKCI as a potential biomarker for CCOC, the PRKCI copy number was determined by qPCR on gDNA extracted from 27 pairs of normal (peripheral blood cell DNA) and CCOC tumor specimens. Elevated PRKCI copy number was found in 20 out of 27 CCOC tumor specimens when compared with peripheral blood cell DNA as normal reference (Figure 2D). Conversely, microarray expression profiling of 10 human CCOC specimens indicated an average of 6.70-fold up-regulation of PRKCI mRNA when compared with 10 normal ovarian surface epithelium samples (Figure 2E). qRT-PCR further validated PRKCI overexpression in CCOC tumor specimens over normal ovarian surface epithelium samples (Figure 2E). These data implicate amplification and overexpression of PRKCI are less likely artifacts during the establishment of cell lines but real genomic aberrances underlying the tumorigenesis of CCOC.

To delineate the potential prognostic impact of CCOC, an independent set of 40 CCOC tumor specimens (with clinical annotation summarized in Supplementary Table S3) were tested for PRKCI protein level by immunohistochemistry (Figure 2F). Kaplan-Meier analysis of the IHC scores using median cut-off revealed a significantly negative impact of PRKCI expression on overall survival (*p*-value = 0.0309, log-rank test) (Figure 2G). Consistent observation can also be obtained by Cox regression, which considers the IHC score as continuous variable and thus avoids subjective cut-off. As per increment of the IHC score of PRKCI by 1, an increase of hazard by 1.21-fold is suggested with statistical significance (95% CI: 1.019-1.44; *p*-value = 0.0274, likelihood ratio test).

### PRKCI regulates the oncogenic properties of CCOC cells *in vitro* and *in vivo*

**Figure 3:**
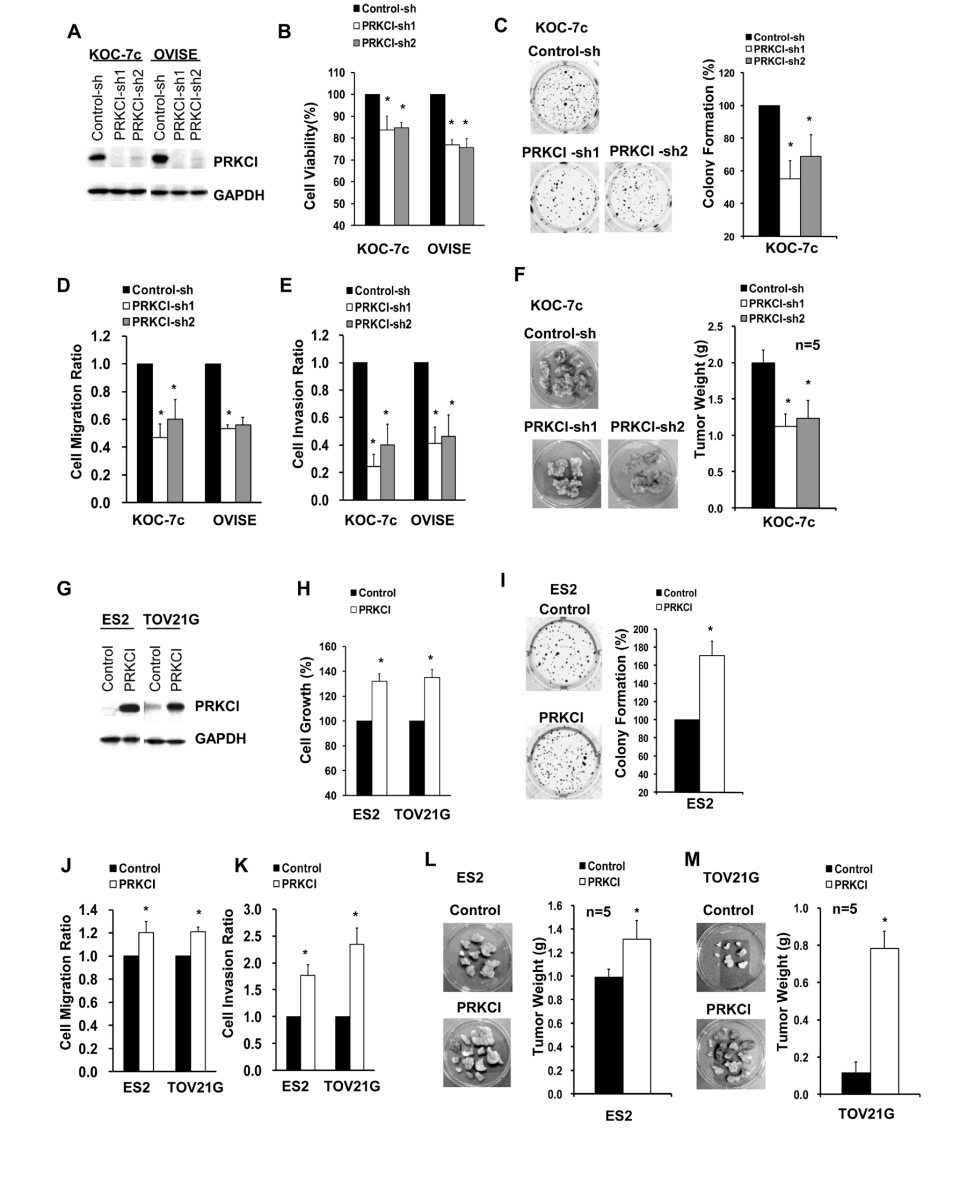
Oncogenic function of PRKCI *in vitro* and *in vivo* **A.** Western blot analysis for PRKCI protein levels in stable PRKCI-knockdown cell lines (KOC-7c and OVISE cells). The stable PRKCI-knockdown cells were tested for **B.** cell viability by CellTiter Blue cell viability assay, **C.** anchorage-independent growth by soft-agar colony formation assay and **D.**-**E.** cell migration and invasion ability by transwell-based cell migration and invasion assays. **F.** The cells were intraperitoneally injected into athymic nude mice (5 mice per group). The mice were allowed to grow for 3-6 weeks. After that, xenograft tumor samples were collected and average tumor weight of different groups of mice were measured. **G.** Western blot analysis for PRKCI protein levels in stable PRKCI-overexpressing cell lines (ES2 and TOV21G cells). The stable PRKCI-overexpressing cells were tested for **H.** cell growth by CellTiter Blue cell viability assay, **I.** anchorage-independent growth by soft-agar colony formation assay and **J.**-**K.** cell migration and invasion ability by transwell-based cell migration and invasion assays. **L.** The cells were intraperitoneally injected into athymic nude mice (5 mice per group). The mice were allowed to grow for 3-6 weeks. After that, xenograft tumor samples were collected and average tumor weight of different groups of mice were measured. % cell growth, % colony formation, cell migration ratio and cell invasion ratio were calculated relative to the corresponding control cells. *p < 0.05.

The biological function of PRKCI was investigated by knockdown and overexpression in CCOC cell lines. Knockdown of PRKCI in KOC-7c and OVISE cells (which have high endogenous PRKCI levels) (Figure 3A), demonstrated reduced *in vitro* cell growth (Figure 3B), non-adherent cell growth (Figure 3C), cell migration (Figure 3D) and cell invasion (Figure 3E). In addition, we have investigated the effect of PRKCI on CCOC *in vivo* tumor growth, using an orthotopic mouse model. Mice with KOC-7c PRKCI-knockdown cells showed significantly smaller tumor masses than mice with KOC-7c control cells (Figure 3F). To further validate the biologic function of PRKCI, ES2 and TOV21G cells, which have low endogenous PRKCI level, were engineered to overexpress PRKCI (Figure 3G). PRKCI-overexpressing ES2 and TOV21G cells were found to have increased *in vitro* cell growth (Figure 3H), non-adherent cell growth (Figure 3I), cell migration (Figure 3J) and invasion (Figure 3K). In addition, mice with PRKCI-overexpressing ES2 and TOV21G cells showed significantly larger tumor masses than mice with ES2 and TOV21G control cells (Figure 3L and 3M).

### The PRKCI inhibitor, sodium aurothiomalate, suppresses the oncogenic function of PRKCI *in vitro* and *in vivo*

**Figure 4:**
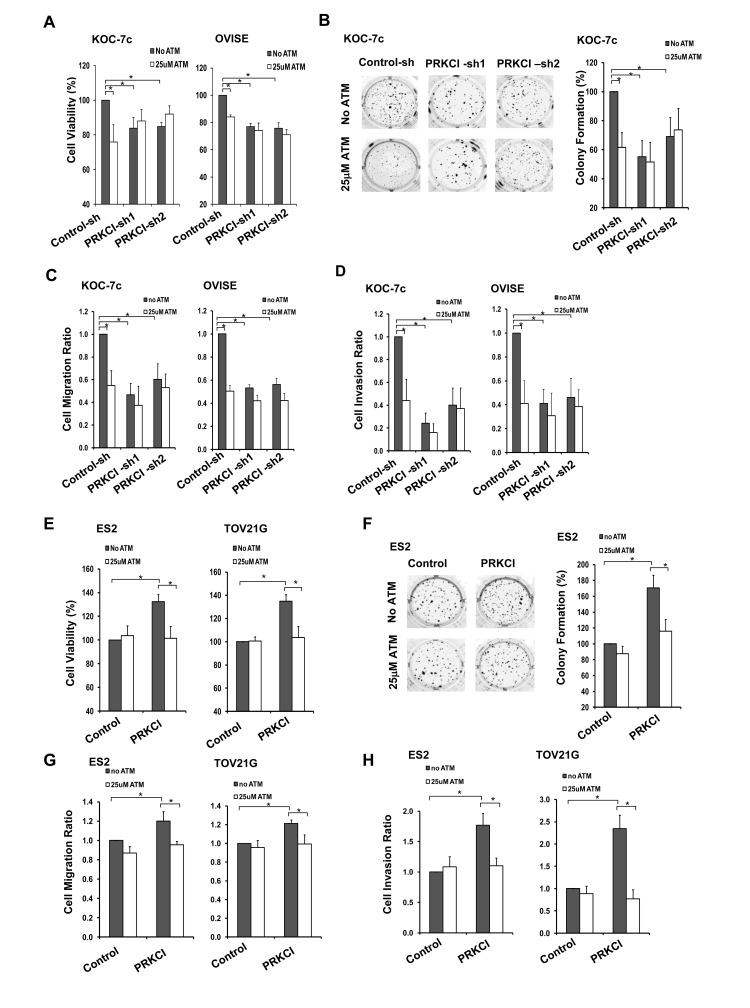
ATM suppression on oncogenic function of PRKCI *in vitro* Stable PRKCI-knockdown cells (KOC-7c and OVISE) were treated with 25µM ATM and were tested for **A.** cell growth by CellTiter Blue cell viability assay, **B.** anchorage-independent growth by soft-agar colony formation assay and **C.**-**D.** cell migration and invasion ability by transwell-based cell migration and invasion assays. Stable PRKCI-overexpressing cells (ES2 and TOV21G) were treated with 25µM ATM and were tested for **E.** cell growth by CellTiter Blue cell viability assay, **F.** anchorage-independent growth by soft-agar colony formation assay and **G.**-**H.** cell migration and invasion ability by transwell-based cell migration and invasion assays. % cell growth, % colony formation, cell migration ratio and cell invasion ratio were calculated relative to the corresponding control cells. *p < 0.05.

A known PRKCI inhibitor, sodium aurothiomalate (ATM), was utilized to validate the biological functions of PRKCI *in vitro* and investigate its potential use *in vivo.* Administration of 25 µM ATM significantly suppressed adherent cell growth on CCOC cells with high PRKCI level, including KOC-7c and OVISE control cells and PRKCI-overexpressing ES2 and TOV21G cells (Figure 4A and 4E). ATM was also found to suppress cell migration and invasion of PRKCI-overexpressing cells. Treatment with ATM on KOC-7c and OVISE control cells and PRKCI overexpressing ES2 and TOV21G cells significantly suppress cell migration and invasion (Figure 4C, 4D, 4G and 4H). Moreover, ATM inhibited non-adherent cell growth of KOC-7c control cells and PRKCI overexpressing ES2 cells by 40% (Figure 4B and 4F).

**Figure 5:**
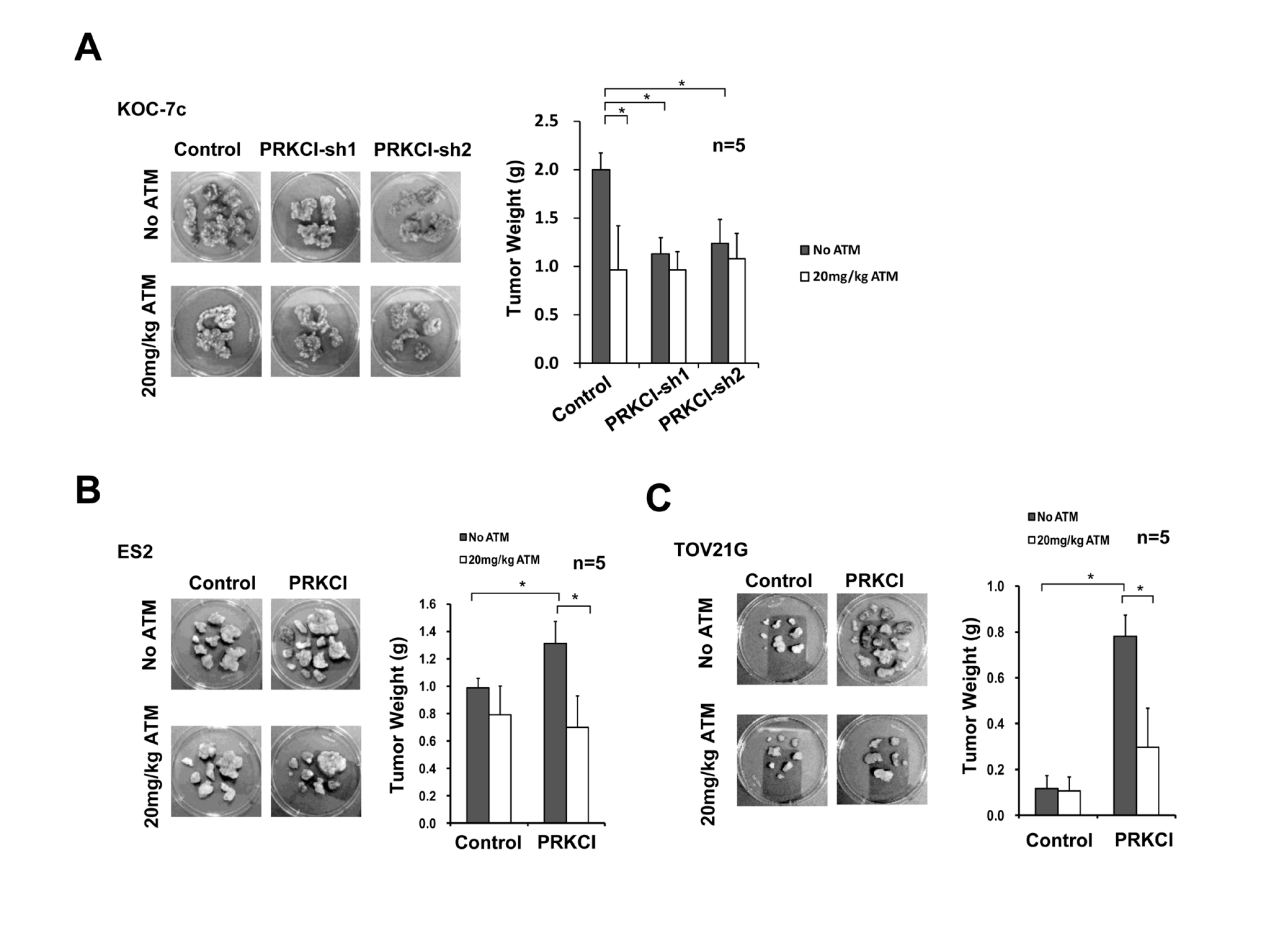
ATM suppression on oncogenic function of PRKCI *in vivo* **A.** PRKCI-knockdown KOC-7c cells, **B.** PRKCI-overexpressing ES2 cells and **C.** PRKCI-overexpressing TOV21G cells were intraperitoneally injected into athymic nude mice (10 mice per group). The mice were allowed to grow for 1-3 week for tumor formation. After that, each group of mice were randomly divided into two (5 mice per group) for ATM treatment. Mice in the ATM treatment groups were intramuscularly injected with 20mg/kg/day of ATM for 14 consecutive days. Mice in the control groups were intramuscularly injected with equal volume of 1X PBS solution. After ATM treatment, mice were sacrificed and xenograft tumor samples were collected. Representative photos of xenograft tumor samples were shown. Average tumor weight of each group was shown in the bar chart at the right panel. *p < 0.05.

In order to determine the effect of ATM on *in vivo* tumor growth, PRKCI-knockdown and PRKCI-overexpressing CCOC cells were intraperitoneally injected into athymic nude mice and the mice were administrated with 20 mg/kg/day for 14 consecutive days. ATM treatment was found to suppress tumor growth in mice with PRKCI overexpressing cells, including mice with KOC-7c control cells (Figure 5A) and PRKCI overexpressing ES2 and TOV21G cells (Figure 5B and 5C). The results indicated the potential use of ATM in the treatment of PRKCI overexpressed clear cell ovarian cancer.

### MEK/ERK and Akt signaling pathways mediate the oncogenic effect of PRKCI

**Figure 6:**
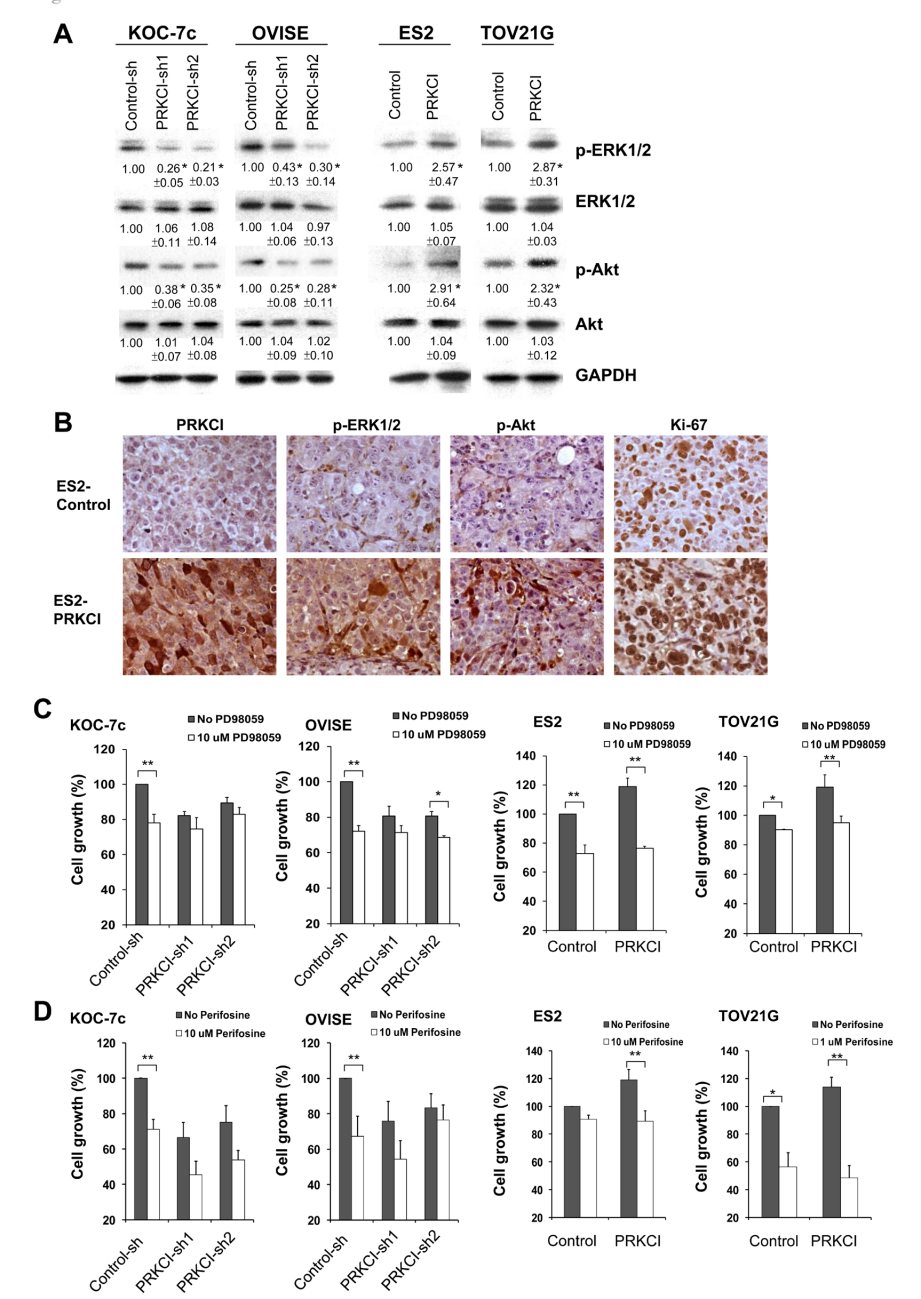
PRKCI regulates oncogenic function through the activation of the ERK and Akt signaling pathways **A.** Activities of ERK signaling and Akt signaling in the PRKCI-knockdown cells (KOC-7c and OVISE) and PRKCI-overexpressing cells (ES2 and TOV21G). The number beneath the band was the relative expression level of that protein. It was calculated first by normalizing with GAPDH and then in relation to the expression of the corresponding control sample. The results shown above are the representative one of three independent experiments. *p < 0.05. **B.** Immunohistochemistry staining of xenografts derived from ES2 PRKCI overexpressing or ES2 control cell lines. Typical sections stained for PRKCI, phospho-ERK, Phospho-Akt and Ki-67 were shown. Cell growth of PRKCI-knockdown cells (KOC-7c and OVISE) and PRKCI-overexpressing cells (ES2 and TOV21G) with the treatment of **C.** PD98059 and **D.** perifosine were determined by CellTiter Blue cell viability assay.

To better understand the mechanisms for PRKCI induced biological function of CCOC cells, downstream signaling pathways of PRKCI in CCOC cells were investigated. MEK/ERK and Akt signaling pathway was found to be activated by PRKCI using western blot analysis of knock-down and overexpressing CCOC cell lines (Figure 6A). MEK/ERK and Akt signaling pathways have higher activity in PRKCI overexpressing cells as demonstrated by higher levels of pERK/pAKT. IHC staining of a xenograft of ES2 PRKCI-overexpressing cells demonstrated elevated levels of PRKCI and higher levels of phospho-ERK1/2, phospho-Akt and Ki-67 when compared with xenograft derived from ES2 control cell line (Figure 6B). The activation of MEK/ERK and Akt signaling pathways is required for PRKCI inducing tumor growth as tumor growth in PRKCI overexpressing cells were significantly suppressed by the MEK/ERK and Akt inhibitors (PD98059 and perifosine) (Figure 6C and 6D).

## DISCUSSION

Clear cell ovarian cancer is an aggressive subtype of ovarian cancer that is less responsive to chemotherapy and has a poorer prognosis. Unfortunately, the genomic basis for this tumor remains unknown. In this study, we integrated genome-wide aCGH and microarray analysis to identify amplified DNA and overexpressed genes (mRNA) in CCOC. The gene list was filtered using PathwayStudio software to identify genes with cancer-related biological function. The 19 genes identified are enriched for potential ‘driver” genes whose function is important for the biology and clinical features of the tumor. In addition, “driver” genes can be potential therapeutic targets. This approach of an integrated genomic analysis has been shown to be effective at identifying novel targets in other tumors [16-19].

Our integrated genomic approach has clear limitations in that it could underestimate the number of “driver’ genes in CCOCs. Recently, a comprehensive study interrogating the robustness of 12 aCGH platforms has revealed aCGH may miss 20-70% of CNV events when manufacturer recommended bioinformatics workflow is conducted (as performed in this study) [20]. In this regard, our aCGH analysis may miss important amplified genes as evidenced by the fact that qPCR further detected the amplification of PRKCI in three additional cell lines KOC-7c, SMOV2 and HAC2 (Supplementary Figure 1). In addition, we integrated expression profiles from CCOC specimens that were unmatched to those we analyzed for CNV, which may introduce bias that will underestimate the amplified genes, which are overexpressed. However, while this algorithm may miss some “driver” genes, its filtering process increases the probability that the genes identified will be important for the biology and clinical features of CCOC. Secondly, ovarian surface epithelia were used in the transcriptomic study to identify genes that are potentially highly expressed in CCOC. The identification of precursor(s) of epithelial ovarian carcinoma as a highly heterogeneous entity remains a highly-debated topic, implicating potential limitations of our experimental design. Recent studies have linked ovarian endometriosis as a putative origin of CCOC, but the pathogenesis has been largely undefined [21]. Unfortunately, whole transcriptomic profiling study of ovarian endometriosis still remain scanty. In a recently published study interrogating 10 human ovarian endometriosis *versus* normal uterine endometrium, significant upregulation was observed for PRKCI in ovarian endometriosis (1.81-fold, *p* = 0.0001) [22]. When compared to our CCOC data (since both datasets were based on the same array platform Affymetrix Human Genome U133 Plus 2.0), PRKCI was found significantly overexpressed in CCOC compared to ovarian endometriosis (3.82-fold, *p* = 0.0021, Supplementary Figure 2). These data further implicate the participation of PRKCI in the onset of CCOC and warrant additional studies using larger patient cohorts.

As proof of principle, we analyzed in detail the function of PRKCI in CCOC progression, which is one of the most frequently amplified and highly overexpressed genes. Until recently, the biological significance of PRKCI in clear cell ovarian carcinoma remains largely unknown. Nevertheless, integrated genomic and phenotypical analyses of PRKCI in CCOC clinical specimens have suggested PRKCI has the hallmarks of a driver gene in CCOC (Figure 2-4). PRKCI gene locates on chromosome 3q26, which contains several known oncogenes, including *PIK3CA*, *SOX2* and *ECT2*. Amplification of 3q26 is one of the most prominent copy number variation features that potentially drives the tumorigenesis in several types of solid tumors [23], including high-grade serous ovarian cancer [24]. However, subsequent functional analyses by ectopic overexpression of PRKCI in serous ovarian cancer cell lines failed to alter the proliferation, colony formation or chemoresistance [24]. Rather, PRKCI overexpression leads to transformation of ovarian surface epithelial cells, a potential precursor for a subset of epithelial ovarian carcinoma [21]. Conversely, high PRKCI expression level correlated with poor clinical outcome of CCOC patients and expression of PRKCI drives multiple important biologic properties including tumor cell migration, invasion and *in vivo* growth. Moreover, the clinical relevance of PRKCI in CCOC is further implicated by its specific small molecule inhibitor sodium aurothiomalate (ATM), which efficiently abrogates the oncogenic effects of PRKCI in CCOC cell lines *in vitro* and *in vivo*.

The biological significance of PRKCI in CCOC is further suggested by the distinctive molecular context of this ovarian cancer subtype. PRKCI represents a structurally and functionally distinct subclass in the protein kinase C family which is less responsive to receptor tyrosine kinase or G-protein coupled receptor initiated diacylglycerol or calcium signals. One of the major second massagers activating PRKCI is phosphatidylinositol 3,4,5-trisphosphate (PIP3), the downstream product of PI3 kinase [25], through specific protein-protein interactions mediated by its unique N-terminal Phox-Bem1 (PB1) domain [26]. Furthermore, PIP3 creates the docking point for the activation of 3-phosphoinositide dependent protein kinase-1 (PDK-1), resulting in priming phosphorylation of PRKCI at T555 to further enhance its kinase activity [27]. Interestingly, more than 50% of CCOCs are characterized by activating mutations of *PI3KCA* or deletion of *PTEN*, both of which lead to excessive generation of PIP3 to facilitate PRKCI mediated biology. It is thus hypothesized that a CCOC clone with PRKCI amplification and overexpression may be more favored in clonogenic selection for its tumorigenic predisposition.

The PRKCI inhibitor ATM binds to the Cys-69 of PRKCI. Cys-69 a unique amino acid residue found in the PB1 domain of PRKCI and binding of ATM to Cys-69 of PRKCI inhibits PRKCI from interacting with Par-6 and p62. ATM specifically inhibits protein-protein interaction of PRKCI but not other PB1 domain containing proteins, including p62-p62, p62-NBR1 and MEKK3-MEK5 interactions [28]. ATM can specifically suppress tumor growth in PRKCI overexpressing CCOC cells. Of interest, ATM has been used for treating rheumatoid arthritis and is in a Phase 1 clinical trial of human non-small cell lung cancer [29, 30]. It would be reasonable to consider a clinical trial identifying patients with PRKCI copy number amplification and mRNA overexpression in CCOC patients and treating them with ATM.

This study is the first report of an integrated genomic analysis of CCOC through which we have identified for the first time PRKCI as a potential target gene in human clear cell ovarian cancer. PRKCI was copy number amplified and mRNA overexpressed in CCOC cell lines and patient specimens. Higher PRKCI levels were associated with increase in cell proliferation, metastatic potential and tumorigenicity of CCOC cells. These findings also support the potential use of PRKCI inhibitor, ATM, as a therapeutic agent to improve the treatment of human clear cell ovarian cancer.

## MATERIALS AND METHODS

### Cell lines culture

The human CCOC cell lines KOC-7c, OVISE, OVSAYO, ES-2, TOV21G, RMG1, KK, OVMANA, SMOV2, HAC2, OVTOKO and OVAS were maintained in RPMI 1640 medium supplemented (Lonza, Hopkinton, MA) with 10% FBS, 1% L-Glutamine and 1% penicillin/streptomycin (Invitrogen Life Technologies, Inc. Carlsbad, CA). Immortalized ovarian surface epithelial (OSE) cell lines, IOSE-80 and IOSE-120, were maintained in a 1:1 mixture of medium 199 (Invitrogen Life Technologies, Inc. Carlsbad, CA) and medium 105 (Sigma, St. Louis, MO), supplemented with 10% fetal bovine serum (FBS) and 1% L-glutamine. ES-2, TOV21G and RMG1 cell lines were purchased from American Type Culture Collection. KOC-7c, OVISE, OVSAYO, KK, OVMANA, SMOV2, HAC2, OVTOKO and OVAS were kindly provided by Professor Hiroaki Itamochi at Tottori University School of Medicine, Japan.

### Stable cell lines preparation

The establishment of stable cell lines was reported in previous publication [11]. Briefly, ectopic overexpression and knockdown of PRKCI were performed by lentiviral infection of PRKCI cDNA carrying pLoc expression vector (pLoc vector series) and PRKCI shRNA carrying vectors (pLKO vector series) respectively. For the knockdown of PRKCI, two PRKCI specific shRNA vectors were employed and the shRNA target sequences are AGTACTGTTGGTTCGATTAAA (PRKCI-sh1) and AGTACTGTTGGTTCGATTAAA (PRKCI-sh2). PRKCI expression / shRNA vectors, control vectors and lentiviral packaging system (Thermo Scientific, Tewksbury, MA) were transfected into 293T cells with X-tremeGENE (Roche, Branford, CT) for viral production. Cells infected with scramble control shRNA served as a control. Stably infected cells were selected and maintained in puromycin containing medium. CCOC cell lines ES2 and TOV21G were infected with PRKCI expression vector (pLoc-PRKCI) for overepression of the gene. Cells infected with RFP expression vector (pLoc-RFP) served as the control. Stably infected cells were selected and maintained in blasticidin S containing medium. Clones and primary cell lines were used in cell viability, soft agar, cell migration/invasion assay *in vitro* assays previously described [11].

### Tissue specimens

27 paired normal (Buffy coat genomic DNA obtained from the same patient) and clear cell ovarian tumor genomic DNA specimen for copy number variation analysis were obtained from GOG-0136. Detailed information of GOG-0136 is available at the NCI webpage (https://clinicaltrials.gov/ct2/show/NCT00897442?term=GOG+0136&rank=1). 10 microdissected CCOC specimens and 10 normal ovarian surface epithelium (OSE) cytobrushing specimens for RNA extraction and microarray analysis were obtained from primary tumors of untreated ovarian cancer patients and healthy donors respectively at the Brigham and Women’s Hospital (Boston, MA). 40 FFPE tumor specimens from patients with clear cell ovarian cancer for immunohistochemical analysis of PRKCI and Kaplan-Meier analysis of patient survival according to PRKCI expression level were obtained from Tottori University School of Medicine (Tottori, Japan).

### Array-based comparative genomic hybridization

Detailed procedure of aCGH hybridization and data analysis have been reported previously [13]. In brief, genomic DNA from 12 human CCOC cell lines were extracted and profiled on the Agilent 105k Human Genome CGH Microarray according to manufacturer’s instructions. Genomic coordinates for this array are based on the NCBI build 36, March 2006 freeze of the assembled human genome (UCSC hg18), available through the UCSC Genome Browser. Data were subsequently imported into CGH Analytics software (version 3.4.40, Agilent Technologies) for visualization. GISTIC analysis was performed to identify disease related amplification and deletion chromosomal regions.

### Microarray analysis

RNA of 12 human CCOC cell lines, 10 human CCOC specimen and 10 normal ovarian surface epithelium specimen were extracted and microarray analysis was performed as described protocol [12]. In brief, following a single strand reverse transcriptase and amplification step, samples were hybridized to Affymetrix U133 Plus 2.0 microarrays. Class comparison in BRB ArrayTools version 3.2.2 software was utilized to identify differentially expressed genes.

### Quantitative PCR and RT-PCR

DNA and RNA samples of 12 human CCOC cell lines were subjected to qPCR and RT-PCR for analysis of PRKCI amplification and overexpression using an iCycler iQ Real-Time PCR Detection System (Bio-Rad, Hercules, CA). PRKCI copy number variation qPCR was performed with iQ SYBR Green Supermix (Bio-Rad, Hercules, CA) according to manufacturer’s protocol using the following primers: Forward: 5’-GAGAGACTCGCCTCCTGAG-3’ and Reverse: 5’-ACTCGCAACCCCAAATACACA-3’. DNA content was normalized to that of Line-1, a repetitive element for which copy numbers are similar between normal and neoplastic cells. Relative DNA copy number was determined by normalizing to that of pooled normal gDNA sample. PRKCI mRNA expression qRT-PCR was performed with the One-Step qRT-PCR with SYBR Green kit (Invitrogen Life Technologies, Inc., Carlsbad, CA) using the primers: Forward: 5’-TACGGCCAGGAGATACAACC-3’ and Reverse: 5’-AAGAGCCCACCAGTCAACAC-3’. mRNA content was normalized to that of the housekeeping genes GAPDH, GUSB, and cyclophillin. Relative mRNA expression was determined by normalizing to that of the average of the two immortalized OSE cell lines. Relative expression of PRKCI was determined by comparing the expression levels of CCOC lines with those in the 2 normal OSE cell lines.

### Immunohistochemistry

Immunochemistry (IHC) on FFPE xenografts and patient tumor specimen were performed using primary antibodies against human PRKCI (Abcam, USA), human phospho-ERK1/2 (Cell Signaling Technology, USA) and human Ki-67 (Sigma, USA) as described [31].

PRKCI protein expression grading was conducted by determining the percentage and intensity of positive cells in 3 different areas at low-power magnification. First, the percentage of positive cells in each section was scored with a 5-point scale: 0 for < 5%, 0.25 for 5%-25%, 0.5 for 26%-50%, 0.75 for 50%-75%, and 1 for over 75%. Second, the intensity of positive signal was scored with a 3-point scale: 1 for weak staining, 2 for moderate staining, and 3 for intense staining. The weighed score of PRKCI staining intensity of each section was obtained by multiplying the percentage score by the intensity score (the maximum weighed score is 4). The IHC quantification was performed by 2 individuals, including 1 pathologist for independent IHC scoring. The average score was used for downstream analyses. The scoring was done blinded to the clinical data.

### *In vivo* orthotopic mouse model

Female 4-6 weeks old athymic nude mice were purchased from Charles River Laboratories. CCOC cells were intraperitoneally injected into mice (For each mouse, 1 x 10^6^ ES2 cells, 4 x 10^6^ TOV21G cells or 5 x 10^6^ KOC-7c cells were injected). Mice were sacrificed 3-6 weeks after tumor cell injection. Sodium aurothiomalate (ATM) dissolved in 1X PBS was administrated intramuscularly at the thigh of the mice starting from 1-3 week after injection of CCOC cells at a dose of 20 mg/kg/day for 14 consecutive days. Equal volume of 1X PBS solution was employed as control. Mice were sacrificed after treatment. In all *in vivo* experiments, tumor burden was assessed by the total tumor weight at the time of sacrifice.

## SUPPLEMENTARY MATERIALS FIGURES AND TABLES



## Abbreviations

The abbreviations used are:

CCOC: clear cell ovarian cancer
SOC: serous ovarian cancer
ATM: sodium aurothiomalate
CNV: copy number variation
aCGH: array comparative genomic hybridization
GISTIC: Genomic Identification of Significant Targets In Cancer
OSE: ovarian surface epithelium
qRT-PCR: quantitative real-time polymerase chain reaction
TCGA: The Cancer Genome Atlas
