# Supplementary material for: Integrated genomic analysis of clear cell ovarian cancers identified PRKCI as a potential therapeutic target
Source: Oncotarget. 2017 Aug 4;8(57):96482–95. doi: 10.18632/oncotarget.19946 (PMC5722499; doi:10.18632/oncotarget.19946)
Supplement: Supplementary file 1 [file oncotarget-08-96482-s001.pdf]

Integrated genomic analysis of clear cell ovarian cancers identified PRKCI as a potential therapeutic target

SUPPLEMENTARY MATERIALS

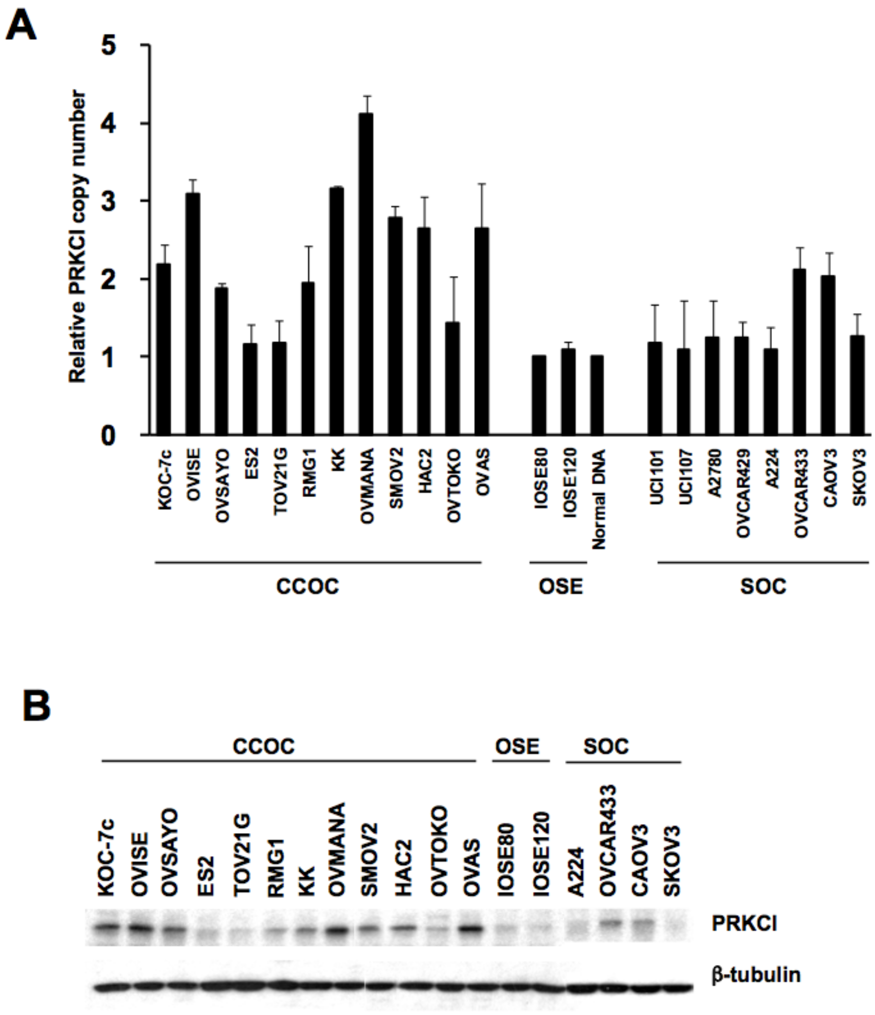

Supplementary Figure 1: Comparison of the DNA copy number and protein expression level of PRKCI in clear cell ovarian cancer (CCOC) cell lines, serous ovarian cancer (SOC) cell lines and ovarian surface epithelial (OSE) cell lines. (A) Relative DNA copy number of PRKCI as determined by qPCR. (B) Protein levels of PRKCI as determined by Western blot.

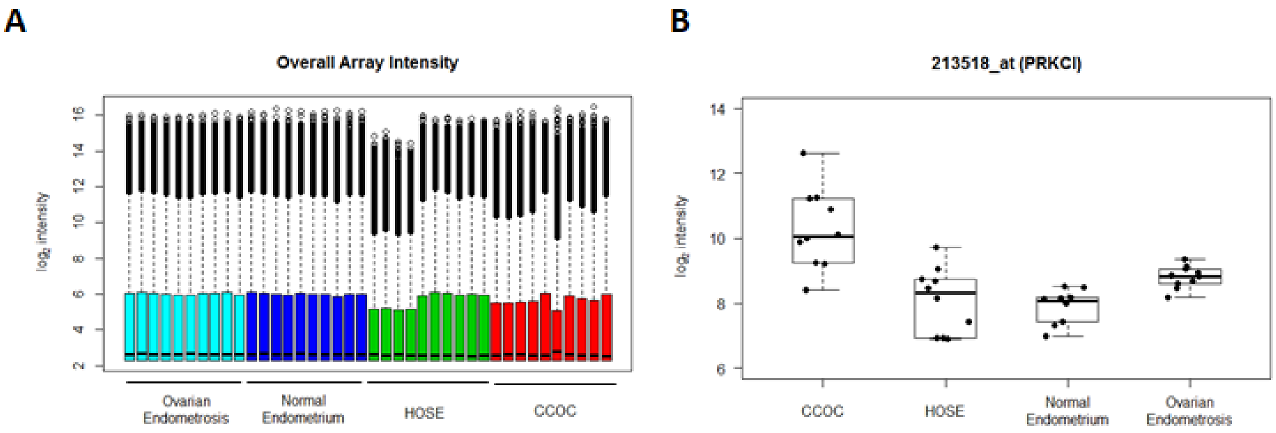

Supplementary Figure 2: Comparison of the PRKCI expression level in CCOC, ovarian epithelial cells (HOSE), normal endometrium and ovarian endometriosis. Microarray data for endometrium and endometriosis were retrieved from NCBI's Gene Expression Omnibus (https://www.ncbi.nlm.nih.gov/geo/) with the accession number of GSE7305. Raw CEL files of all samples were subjected to quantile normalization using R/Bioconductor package *gcrma*. (A) Overall array intensity after normalization suggests no significant interstudy bias. (B) Boxplot showing the level of PRKCI expression as log2 probe intensity. PRKCI is significantly overexpressed in CCOC compared to other three tissue types at fold-changes of 5.22, 6.91 and 3.82 respectively (p-value: 4.87×10^{-4}, 4.33×10^{-5} and 2.08×10^{-3}, by Mann-Whitney-Wilcoxon test).

Supplementary Table S1. Amplification peaks identified by GISTIC.

Amplification peaks	Peak Limits	q value	Frequency	Number of genes in peak
**1q44**	chr1:233113008-246874991	0.13427	5/12	95
**2q13**	chr2:113611779-113911005	0.0008108	5/12	2
**3q26.31**	chr3:169954219-178523082	0.22886	6/12	30
**4p16.3**	chr4:1532183-2129305	0.10152	3/12	11
**4q32.1**	chr4:159421407-159792448	0.011976	1/12	1
**6q26**	chr6:161390383-162077009	0.23303	1/12	3
**8q11.23**	chr8:43647064-55533891	0.021335	5/12	22
**8q24.3**	chr8:138686236-141886722	0.059432	5/12	8
**10q24.1**	chr10:98847154-99432221	0.05277	5/12	16
**11q13.3**	chr11:70081659-70971957	0.018447	3/12	8
**11q22.1**	chr11:99716458-101536280	0.020398	3/12	8
**12q24.31**	chr12:123934406-124115384	0.05277	6/12	3
**17q22**	chr17:53582424-56121846	0.00034	7/12	40
**19q12**	chr19:33432676-44172883	0.010038	5/12	130
**20q11.22**	chr20:33059672-33669568	0.0088789	7/12	11
**20q13.2**	chr20:51268052-51994180	8.34E-06	10/12	3

Supplementary Table S2. Genes with copy number amplification and overexpression.

Amplification peak	Amplified and overexpressed genes
1q44	RBM34, ARID4B, ARID4B, MTR, FH, ADSS, PPPDE1, HNRNPU
2q13	PSD4, PAX8
3q26.31	ECT2, PRKCI, GPR160, MYNN
4p16.3	TMEM129, FGFR3, WHSC1
8q11.23	PRKDC, MCM4, UBE2V2, PCMTD1, RB1CC1, ATP6V1H, LYPLA1, MRPL15
8q24.3	PTK2
12q24.31	BRI3BP
17q22	MKS1, MTMR4, PRR11, PTRH2, TMEM49, TUBD1, APPBP2
19q12	CEBPG, LSM14A, GPI, COX6B1, PSENEN, LIN37, ZNF146, ZNF566, ECH1
20q11.22	EIF6
20q13.2	ZNF217

Supplementary Table S3. Relationships between PRKCI expression and clinicopathological parameters in 40 CCOC patients.

Variable	Case (n = 40)	PRKCI expression Low, No. (%), (n = 16)	PRKCI expression High, No. (%), (n = 24)	*P*-value [a]
**Age**				
<50	11	5 (31.2)	6 (25.0)	0.6645
≥50	29	11 (68.8)	18 (75.0)	
**FIGO stage**				
I + II	31	14 (87.5)	17 (70.8)	0.2162
III + IV	9	2 (12.5)	7 (29.2)	
**T stage**				
T1	27	14 (87.5)	13 (54.2)	0.0876
T2	6	1 (6.25)	5 (20.8)	
T3	7	1 (6.25)	6 (25.0)	
**Regional lymph nodes** [b]				
N0	32	13 (92.9)	19 (90.5)	0.8053
N1	3	1 (7.14)	2 (9.52)	
**Distant metastasis** [c]				
M0	37	14 (100)	23 (95.8)	0.4389
M1	1	0 (0.00)	1 (4.17)	

[a] *P*-values were calculated using Pearson's chi-square test. *P*-values were considered significant when < 0.05.
[b] Regional lymph nodes cannot be assessed in 5 cases.
[c] Distant metastasis cannot be assessed in 2 cases.
